# Impact of wet-lab protocols on quality of whole-genome short-read sequences from foodborne microbial pathogens

**DOI:** 10.3389/fmicb.2023.1253362

**Published:** 2023-11-29

**Authors:** Leonie F. Forth, Erik Brinks, Grégoire Denay, Ahmad Fawzy, Stefan Fiedler, Jannika Fuchs, Anne-Catrin Geuthner, Thomas Hankeln, Ekkehard Hiller, Larissa Murr, Henning Petersen, Ralf Reiting, Christian Schäfers, Claudia Schwab, Kathrin Szabo, Andrea Thürmer, Anne Wöhlke, Jennie Fischer, Stefanie Lüth, Michaela Projahn, Kerstin Stingl, Maria Borowiak, Carlus Deneke, Burkhard Malorny, Laura Uelze

**Affiliations:** ^1^Department of Biological Safety, German Federal Institute for Risk Assessment, Berlin, Germany; ^2^Institute of Microbiology and Biotechnology, Max Rubner-Institut, Kiel, Germany; ^3^Chemical and Veterinary Analytical Institute Rhein-Ruhr-Wupper (CVUA-RRW), Krefeld, Germany; ^4^Department of Veterinary Medicine, Hessian State Laboratory, Giessen, Germany; ^5^Department of Medicine and Infectious Diseases, Faculty of Veterinary Medicine, Cairo University, Giza, Egypt; ^6^Method Standardisation, Reference Laboratories, Resistance to Antibiotics, Federal Office of Consumer Protection and Food Safety, Berlin, Germany; ^7^Chemical and Veterinary Analysis Agency Karlsruhe, Karlsruhe, Germany; ^8^Department of Food Safety, State Office for Consumer Protection Saxony-Anhalt, Halle, Germany; ^9^Institute of Organismic and Molecular Evolution, Johannes Gutenberg University Mainz, Mainz, Germany; ^10^StarSEQ GmbH, Mainz, Germany; ^11^Chemical and Veterinary Analysis Agency Stuttgart, Fellbach, Germany; ^12^Bavarian Health and Food Safety Authority, Oberschleißheim, Germany; ^13^Chemical and Veterinary Analytical Institute Ostwestfalen-Lippe, Detmold, Germany; ^14^Hessian State Laboratory, Kassel, Germany; ^15^Institute for Hygiene and Environment, Hamburg, Germany; ^16^Labor Kneißler GmbH & Co. KG, Burglengenfeld, Germany; ^17^Methods Development and Research Infrastructure, Robert Koch-Institut, Berlin, Germany; ^18^Food and Veterinary Institute Braunschweig/Hannover, Lower Saxony State Office for Consumer Protection and Food Safety, Braunschweig, Germany

**Keywords:** interlaboratory study, whole genome sequencing, protocol, quality, *Campylobacter*, *Escherichia*, *Listeria*, *Salmonella*

## Abstract

For successful elucidation of a food-borne infection chain, the availability of high-quality sequencing data from suspected microbial contaminants is a prerequisite. Commonly, those investigations are a joint effort undertaken by different laboratories and institutes. To analyze the extent of variability introduced by differing wet-lab procedures on the quality of the sequence data we conducted an interlaboratory study, involving four bacterial pathogens, which account for the majority of food-related bacterial infections: *Campylobacter* spp., Shiga toxin-producing *Escherichia coli*, *Listeria monocytogenes*, and *Salmonella enterica*. The participants, ranging from German federal research institutes, federal state laboratories to universities and companies, were asked to follow their routine in-house protocols for short-read sequencing of 10 cultures and one isolated bacterial DNA per species. Sequence and assembly quality were then analyzed centrally. Variations within isolate samples were detected with SNP and cgMLST calling. Overall, we found that the quality of Illumina raw sequence data was high with little overall variability, with one exception, attributed to a specific library preparation kit. The variability of Ion Torrent data was higher, independent of the investigated species. For cgMLST and SNP analysis results, we found that technological sequencing artefacts could be reduced by the use of filters, and that SNP analysis was more suited than cgMLST to compare data of different contributors. Regarding the four species, a minority of *Campylobacter* isolate data showed the in comparison highest divergence with regard to sequence type and cgMLST analysis. We additionally compared the assembler SPAdes and SKESA for their performance on the Illumina data sets of the different species and library preparation methods and found overall similar assembly quality metrics and cgMLST statistics.

## 1 Introduction

The last decade has brought great advances in the instruments, methods and bioinformatics tools used for generating and analyzing bacterial whole genome sequences (WGS; Taboada et al., 2017; Besser et al., 2018). Consequently, food-borne outbreak investigations and source attributions are nowadays based on WGS combined with cluster analysis and epidemiological metadata (O’Brien, 2012; Sekse et al., 2017; Pightling et al., 2018; Jagadeesan et al., 2019; NIHR Global Health Research Unit on Genomic Surveillance of AMR, 2020). Enteritis caused by *Campylobacter* spp. is the most common notifiable bacterial disease in Germany, with cases of 80 to 90 infections per 100,000 people (Robert-Koch-Institut, 2019). In 2022, registered foodborne infections in Germany included 43,608 cases with campylobacteriosis, 9,142 cases of salmonellosis, 571 cases of listeriosis and 1,825 cases of enterohaemorrhagic *E. coli* (EHEC) infection according to the German surveillance database, provided by the Robert-Koch institute (Survstat@RKI). Normally causing symptoms like nausea, vomiting, diarrhea, aches and fever that last a couple of days, life-threatening conditions are possible when the bacteria spread to the bloodstream (Yang et al., 2017; Chlebicz and Slizewska, 2018). Infection risk is minimized by correct food and kitchen hygiene (Redmond and Griffith, 2009). In an outbreak, timely and correct source attribution is crucial to minimize the impact on public health. WGS provides the highest level of bacterial strain discrimination and additionally provides information on virulence factors, antimicrobial resistance genes, and their dissemination potential by horizontal gene transfer (EFSA Panel on Biological Hazards et al., 2019). Commonly, a number of laboratories are involved in the investigations of food-related outbreaks, which each employ their own sequencing routine. Therefore, one must consider the possibility that differences in the resulting genome assemblies do not reflect biological variability, but are the consequence of technological artefacts introduced by the applied wet-lab and dry-lab protocols. As the actual genomic sequence is best inferred through high quality raw sequencing data, it is of paramount importance that data of the highest quality is generated and used for the analysis. Although a number of parameters for measuring sequence data quality have been developed (e.g., Q30 base fraction, N50; Van Belkum et al., 2007; Jauhal and Newcomb, 2021), the definition of limits for acceptable data quality is intricate and an on-going process (Gargis et al., 2016; Wagner et al., 2021). Overly rigid numeric quality limits are ultimately not suitable for every purpose and depend, for example, on the sequencing technology or bacterial species. For example, Illumina instruments deliver higher quality bases than Ion Torrent instruments based on their respective innovative technologies for the actual sequencing process (Loman et al., 2012; Carbo et al., 2023), and different coverages may be required depending on the genome complexity of the different species and the intended purpose (Timme et al., 2020b).

Although efforts have been made to standardize wet-lab protocols (Timme et al., 2018, 2020a), different laboratories will always adopt the sequencing devices and methods best suited to their requirements (size of the laboratory / team, schedules, number of sequencing runs, budget restrictions, IT infrastructure). Even if it was possible to oblige investigating laboratories to use identical methods and sequencing devices, it is still unclear which particular method and protocol would generate the best data. Moreover, protocols are regularly optimized when innovations are introduced, such as new technology and software.

Aim of this study was to investigate in depth whether different laboratories with varying protocols and sequencing devices generate comparable sequencing data, suitable for epidemiological investigations and conclusions. In 2019, we performed a pilot study on two DNA samples of *Campylobacter (C.) jejuni*, *Listeria (L.) monocytogenes*, and *Salmonella (S.) enterica* each, which were sequenced by 10 laboratories (Uelze et al., 2020a). In the current study, we included a larger sample panel (10 per species), one additional species [*Escherichia (E.) coli*] and provided participants with the isolate cultures instead of prepared isolate DNA, thus better replicating the practical situation in sequencing of isolates. The interlaboratory study (consisting of two parts, thereafter designated as ring trials part I and II) was conducted in the context of developing an official protocol in frame of the §64 German Food and Feed Code (LFGB) for the whole genome sequencing for typing and characterization of *Salmonella enterica*, *Listeria monocytogenes*, thermophile *Campylobacter* spp., and Shiga toxin-producing and commensal *Escherichia coli* that were isolated from food, feed, food-delivering animals and environmental samples (Bundesamt für Verbraucherschutz und Lebensmittelsicherheit, 2023). In the protocol of the German §64 LFGB (partly species-specific), obligatory and optional quality criteria limits were selected that define acceptable data quality as a reliable ground for epidemiological analysis (Bundesamt für Verbraucherschutz und Lebensmittelsicherheit, 2023), and the adherence to those evaluated in extensive result reports (Uelze, 2021; Forth, 2022). Investigated parameters included Q30 base fraction, assembly coverage depth, contamination control, assembly length, derived genus, number of contigs longer than 1,000 nt and GC content. Additionally, we analyzed further quality characteristics as the N50 and number of full genes, both not included in the official protocol. Here, we present the results for a meaningful selection of sequence and assembly quality characteristics that provide a good view of the overall quality, including Q30 base fraction, contamination control, assembly length and coverage, N50 and full genes, in an open approach independently of any specifications made in the developed §64 LFGB official protocol. We additionally focus on cgMLST and SNP analysis results and differences that can be accounted to specific wet-lab issues.

## 2 Materials and methods

### 2.1 Ring trial set-up

The ring trial was conducted by the National Study Center for Sequencing in Risk assessment, located at the German Federal Institute for Risk Assessment and in the framework of the §64 LFGB working group for NGS bacterial characterization. Participating laboratories included German federal research institutes, federal state laboratories, universities and companies. The ring trial was divided into two parts, based on the species of the bacterial isolates and conducted over the course of 2 years. In the first year, 2021, participants were asked to sequence 10 isolates each of the species *S. enterica* and *L. monocytogenes* (referred to as part I). In 2022, the ring trial was repeated with 10 isolates each of the species *Campylobacter* spp. and Shiga toxin-producing *E. coli* (part II). Some participants completed only one part of the ring trial. In the first ring trial part, 14 laboratories participated. Of these, one of the participants choose to employ two different sequencing devices (and accordingly different library preparation methods), identified as LC05_LS and LC05_S5_LS. In the second ring trial, 15 laboratories participated. An anonymous laboratory identification code was randomly allocated to each participant. Laboratories taking part in both ring trial parts, received differing laboratory identification codes for each year and thus cannot be identified (i.e., LC01 in I ≠ LC01 in II). To differentiate between the participants in the two ring trial parts we added the suffixes LS (for *Listeria* and *Salmonella*) and CE (for *Campylobacter* and *Escherichia*), e.g., LC01_LS and LC01_CE.

Ten bacterial isolates and one DNA sample per species were selected for sequencing (Table 1). In each species-specific dataset, we included two isolates identical to each other (the first two in the sample list, numbered 01 and 02), two times two isolates with a close phylogenetic relationship, one isolate distantly related to the others, two isolates with interesting genetic characteristics (e.g., antibiotic resistance genes, duplicated virulence genes) and one randomly selected isolate. The DNA sample was extracted from a publicly available reference strain in addition to the selected isolates (Table 1). Information about the isolates (apart from the species designation) and DNA sample was not shared with the participants. One participant (LC05_LS in the first part, LC06_CE in the second part) received extracted isolate DNA, due to no available cultivation facilities.

**Table 1:** Overview of samples shipped to the participants in ring trial part I and II.

Species		Matrix	Sample ID	Serovar/Serogroup	MLST
*Salmonella enterica*		DNA	21-RV3-P64-DNA-Salmonella (=ATCC 13076)	Enteritidis	ST11
		Culture	21-RV3-P64-S01	Enteritidis	ST11
			21-RV3-P64-S02	Enteritidis	ST11
			21-RV3-P64-S03	Enteritidis	ST11
			21-RV3-P64-S04	Enteritidis	ST11
			21-RV3-P64-S05	I 4,[5],12:i:-	ST34
			21-RV3-P64-S06	I 4,[5],12:i:-	ST34
			21-RV3-P64-S07	IIIa 41:z4,z23:-	ST2131
			21-RV3-P64-S08	Kentucky	ST198
			21-RV3-P64-S09	Infantis	ST32
			21-RV3-P64-S10	I 4,[5],12:i:-	ST34
*Listeria monocytogenes*		DNA	21-RV3-P64-DNA-Listeria (=ATCC 13932)		ST145
		Culture	21-RV3-P64-L01	IIc	ST9
			21-RV3-P64-L02	IIc	ST9
			21-RV3-P64-L03	IIb	ST5
			21-RV3-P64-L04	IIb	ST5
			21-RV3-P64-L05	IVb	ST6
			21-RV3-P64-L06	IVb	ST6
			21-RV3-P64-L07	IVa	ST20
			21-RV3-P64-L08	IIa	ST26
			21-RV3-P64-L09	IIa	ST121
			21-RV3-P64-L10	IIa	ST37
*Campylobacter*	*jejuni*	DNA	22-RV4-P64-DNA-Campylobacter (=ATCC 33291)		ST2282
	*coli*	Culture	22-RV4-P64-C01		ST1563
	*coli*		22-RV4-P64-C02		ST1563
	*jejuni*		22-RV4-P64-C03		ST21
	*jejuni*		22-RV4-P64-C04		ST21
	*jejuni*		22-RV4-P64-C05		ST61
	*jejuni*		22-RV4-P64-C06		ST61
	*lari*		22-RV4-P64-C07		ST21
	*coli*		22-RV4-P64-C08		ST10187
	*jejuni*		22-RV4-P64-C09		ST4754
	*jejuni*		22-RV4-P64-C10		ST44
*Escherichia coli (STEC)*		DNA	22-RV4-P64-DNA-Escherichia (=EURL strain ED56)	O26	ST21
		Culture	22-RV4-P64-E01	O157	ST11
			22-RV4-P64-E02	O157	ST11
			22-RV4-P64-E03	O103	ST17
			22-RV4-P64-E04	O103	ST17
			22-RV4-P64-E05	O113	ST223
			22-RV4-P64-E06	O113	ST223
			22-RV4-P64-E07	O91	ST11315
			22-RV4-P64-E08	O8	ST162
			22-RV4-P64-E09	O26	ST21
			22-RV4-P64-E10	O128	ST811

Bacterial isolates were obtained from the strain collections of the German national reference laboratories (NRL) for *Salmonella*, *L. monocytogenes*, *E. coli* and *Campylobacter. Salmonella* cryocultures were reactivated on LB agar and incubated overnight at 37°C. The following day, a single colony was picked and inoculated in LB liquid medium and incubated overnight at 37°C under shaking conditions. From the liquid cultures, nutrient high layer agar tubes were inoculated and incubated again overnight at 37°C for shipping. *L. monocytogenes* cryocultures were plated onto Sheep Blood Agar and incubated overnight at 37°C. From the plates, cell material was transferred to Cary-Blair swabs for transportation. Shiga toxin-producing *E. coli* cryocultures were plated on Columbia blood agar plates (Oxoid) and incubated overnight at 37°C. The following day, a 10 μL loop was used to inoculate a stab agar culture using nutrient high layer agar tubes. Tubes were incubated overnight at 37°C. *Campylobacter* spp. cryocultures were plated on Columbia blood agar plates (Oxoid) and incubated at 41.5°C under microaerobic atmosphere (5% O_2_, 10% CO_2_, rest N_2_) for 24 h. A single colony was picked and sub-cultured under similar conditions for 24 h. Cary Blair swabs (Oxoid) were used as transport medium. All plates and tubes were express shipped to the participants.

The participants were asked to follow their established in-house protocols for isolate propagation, DNA extraction, library preparation and sequencing. Information about the employed laboratory procedures and products was captured through an extensive questionnaire. A detailed table including all wet-lab information provided by the participants is included in the Supplementary material (Supplementary Table 1). Raw sequence data files produced by the participants were uploaded to a secure cloud-based platform provided by the National Study Center for Sequencing in Risk assessment. Participants were required to provide their results within 12 weeks after receiving the isolates.

### 2.2 Analyses of sequence data

**Figure 1:**
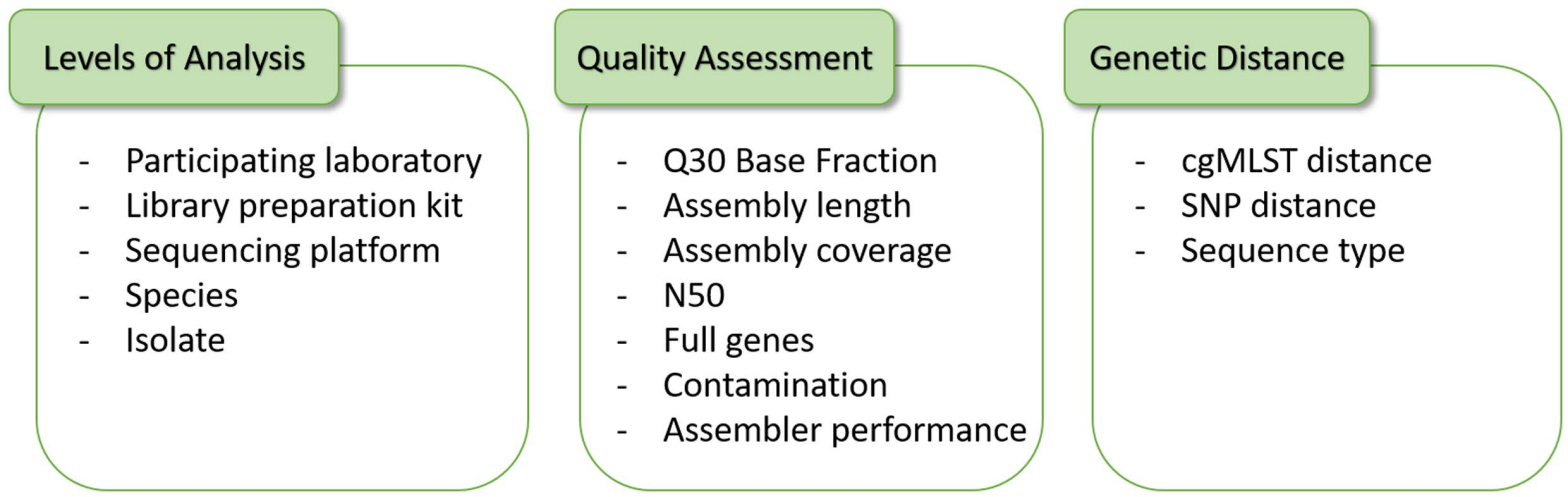
The variance in the data quality and genetic distance can be analyzed on different levels in this study, e.g., grouped per all samples of each participating laboratory, per laboratories applying the same library preparation kit and/or sequencing platform, per species or per isolate.

Overall, 14 participants in ring trial part I (whereof one participant took part with two different sequencing methodologies, thereby counted as 14 + 1) and 15 participants in ring trial part II transmitted sequence data, resulting in a dataset of theoretically 660 samples. As participant LC08_LS encountered difficulties preparing sample 21-RV3-P64-S02, no data was transmitted for this sample, resulting in an overall dataset of 659 samples. A centralized evaluation and assessment of the interlaboratory study results was performed by the organizers of the ring trial, the National Study Center for Sequencing in Risk Assessment. For data interpretation, results were analyzed in regard to different levels, e.g., according to the participating lab, library kit, sequencing platform, species or isolate, each perspective providing insights from a different angle (Figure 1).

All data was subjected to a uniform data analysis with the bioinformatics pipeline AQUAMIS v1.3.8 and v1.3.9 on the raw sequence data with default parameters. AQUAMIS is a Snakemake pipeline and performs all steps of a primary sequence analysis (Deneke et al., 2021a), consisting of read trimming and read quality control (both based on fastp v0.22.0), taxonomic classification (kraken2 v2.1.2), *de-novo* assembly (shovill v1.1.0 including SPAdes), reference identification (mash v2.3), assembly quality control (QUAST v5.0.2 including BUSCO) and contamination detection (confindr v0.7.4). It also determines additional characteristics, e.g., the MLST sequence type based on mlst v2.19.0 and calculates the number of full genes (determined by alignment against a reference sequence as performed in Mash with gene counts determined by QUAST). Ion Torrent data was analyzed using the additional argument “--iontorrent.”

A selection of important quality characteristics was exploratively compared and visualized using R: Q30 base fraction after trimming (percentage of bases with a Phred score ≥ 30), assembly length (calculated as the sum of the length of assembled contigs), average assembly coverage depth [calculated as coverage = (total basepairs after trimming/assembly length)], N50, and amount of full genes. We conducted descriptive and explorative statistical analyses on the quality characteristics to identify major differences between different wet-lab approaches in an unbiased approach.

Sample contamination was identified and quantified based on the AQUAMIS results of confindr and kraken2. Overall, six samples (LC15-22-RV4-P64-C02, -C06 and -C09, LC14-22-RV4-P64-C04, LC02-21-RV3-P64-S03, and LC02-21-RV3-P64-L09) were strongly contaminated. Additional four samples were swapped (LC03-22-RV4-P64-C02, -C03, -C05 and -C06). All 10 samples were therefore excluded from the principal component, cgMLST and SNP analysis.

Principal component analysis was performed on the duplicate isolates of *C. jejuni*, *E. coli*, *L. monocytogenes*, and *S. enterica* in R studio with R v4.2.2 applying the package FactoMineR v2.7. For result visualization the package factoextra v1.0.7 was used.

CgMLST analyses were performed using the ChewieSnake pipeline v3.0 and v3.1.1 (Deneke et al., 2021b), based on chewBBACA v2.0.16 (Silva et al., 2018) in two approaches. First, default settings were used. Later, to analyze the influence of frame shifts on the assemblies, a length filter was applied deploying the additional parameters --remove_frameshifts and --allele_length_threshold 0.05. For *L. monocytogenes*, the Ruppitsch scheme (1,691 loci; Ruppitsch et al., 2015) was derived from the cgMLST.org nomenclature server, for *Salmonella enterica* the Enterobase scheme was applied (http://enterobase.warwick.ac.uk/species/senterica/download_data; 3,000 loci), for *E. coli* the cgMLST scheme of the European Food Safety Authority (EFSA; https://doi.org/10.5281/zenodo.6655441; 2,359 loci) and for *Campylobacter* spp. the Cody scheme (Cody et al., 2017; https://pubmlst.org/bigsdb?db=pubmlst_campylobacter_seqdef&page=schemeInfo&scheme_id=4; 1,343 loci).

The maximum fraction of missing loci was up to 4% for *L. monocytogenes* assemblies, up to 7% for *S. enterica* assemblies, up to 2% for *E. coli* assemblies and up to 8% for *Campylobacter jejuni/coli* assemblies. For *C. lari* assemblies, the fraction of missing loci ranged between 39–42% because the applied Cody scheme is designed on *Campylobacter coli* and *jejuni* assemblies. To our knowledge, there is no common cgMLST scheme for *C. lari*. As the fraction of missing loci for the only *C. lari* isolate is similar between the different participants and the goal is to show differences in the datasets of an identical isolate, we decided to present the information, however, it is emphasized that the resolution is low.

With application of the frameshift filter the following number of loci were not considered in allele calling (minimum/maximum/median): *L. monocytogenes* (15/31/18), *S. enterica* (113/139/118), *E. coli* (44/63/46) and *Campylobacter jejuni/coli* (12/61/18), and *Campylobacter lari* (21/34/23).

To investigate the impact of variability on clustering, cgMLST analyses were performed on the assemblies of the participants’ data for the four species with exclusion of strongly contaminated samples. Median cgMLST allele distance was calculated from the allelic differences of one assembly to other assemblies within identical isolates. For a visualized example of the median calculation see Figure 6 in Uelze et al. (2020a).

SNP analyses were performed on five samples per species individually, including the duplicated isolate, the extracted DNA (control) and two other isolates (numbered 03 and 07), applying the SnippySnake pipeline v1.2.2 based on Snippy v4.6.0 with trimmed read data and an internal reference (assembled genome of participant LC01’s data). The calculation of the number of masked bases (including variants with heterozygous or poor quality genotype), unaligned bases and individual reduction of the core genome is included in the pipeline. The Gubbins filter usually recommended for SNP analyses of *Campylobacter* spp. samples was not applied, due to the close relatedness of the samples.

For comparison of the assembler effect, the assembly was repeated using the AQUAMIS pipeline v1.3.9. with the assembler SKESA (Souvorov et al., 2018), instead of SPAdes (Bankevich et al., 2012), set through an additional parameter (--assembler skesa). Since SKESA does not support the assembly of Ion Torrent data, only Illumina raw data was included in the analysis. The quality metrics were analyzed in direct comparison. The generated assemblies were further subjected to cgMLST analysis with ChewieSnake v3.1.1 to enable the comparison of numeric differences in allele matches. “MATCH” were calculated by adding exact allele matches (EXC) and inferred new alleles (INF) as provided by chewBBACA integrated into ChewieSnake to prevent a bias through sequential analysis. Contaminated and interchanged samples were excluded in the analysis. Overall, data aggregation and visualization were performed in R studio with R v4.2.2 (R Core Team, 2022).

## 3 Results

### 3.1 Used kits and wet-lab procedure

**Figure 2:**
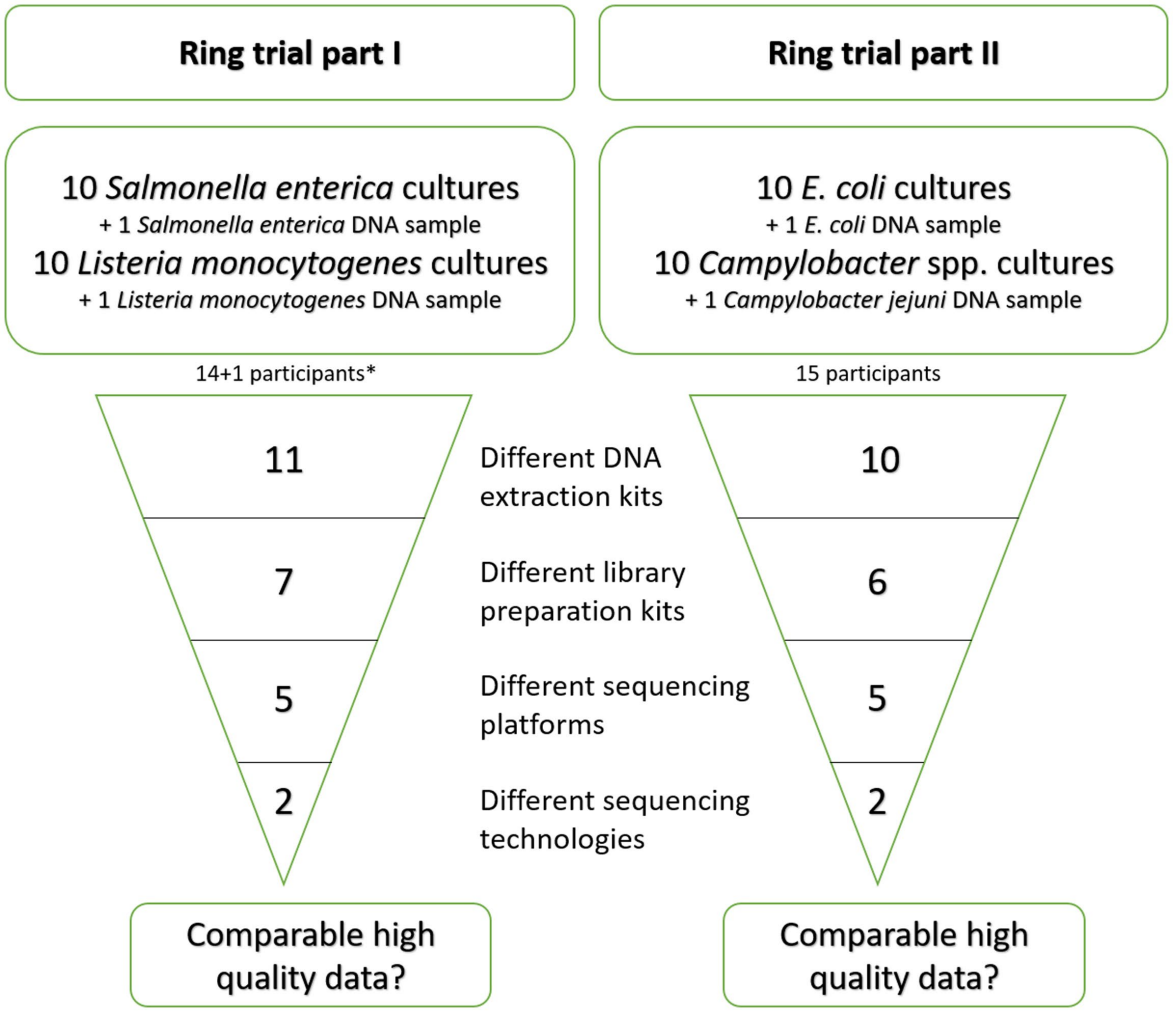
Schematic overview of the differences in the wet-lab preparation kits and methods applied by the participants in the ring trial parts. *: 14 participants overall, whereof one participated with two different sequencing technologies and accordingly two different kits. One participant provided no data on applied kits while reporting the sequencing device.

In the first part of the ring trial, targeting *S. enterica* and *L. monocytogenes* isolates, 14 laboratories participated. As one laboratory participated with two different sequencing technologies and accordingly two different library preparation kits, we will further simplify the total number of participants to 15. Participant LC14_LS provided no metadata besides the applied sequencing device, therefore the calculated values for different methods will add up to 14. In the ring trial part II, targeting Shiga-toxin producing *E. coli* (further referred to as *E. coli*) and *Campylobacter* spp. isolates, 15 different laboratories participated. A number of different DNA extraction and library preparation kits was used by the participants for further sequencing of the libraries on NGS devices (Figure 2, details in Supplementary Table 1). During ring trial part I, 11 different DNA extraction kits were employed. Similarly, 10 different DNA extraction kits were selected during ring trial part II. Only two kits [Invitrogen PureLink Genomic DNA Mini Kit and DNeasy Blood & Tissue Kit (Qiagen)] were used more than once (Supplementary Table 1). A similar diversity was observed in the chosen library preparation kits. In the ring trial part I, seven different kits were used. The majority of participants (8/15) applied the Illumina® DNA Prep, (M) Tagmentation kit (Order No.: 20018704 and 20018705, respectively, for 24 or 96 reactions; Table 2). The remaining participants chose one of the following kits: TruSeq Nano DNA Low Throughput Library Prep Kit, Ion Plus Fragment Library Kit, NEBNext® Ultra™ II FS DNA Library Prep Kit, Nextera XT DNA Library Preparation Kit (96 samples), NEBNext® Ultra™ II DNA Library Prep Kit, NEBNext® Fast DNA Library Prep Set for Ion Torrent. One participant (LC14_S5_LS) did not provide any information regarding the used kits. Some participants reported deviations from the kit protocol, e.g., halving the reagents’ volumes (5 participants, Illumina® DNA Prep, (M) Tagmentation and TruSeq Nano DNA Low Throughput Library Prep Kit), use of 70% instead of 80% ethanol (v/v), PCR-free or several rounds of PCR (Supplementary Table 1). Regarding the DNA fragmentation, both enzymatical (10/15) and mechanical methods (4/15) were employed. When pooling the libraries, the majority of participants weighted them according to the genome size, while two participants (LC06_LS, LC11_LS) included all probes equally weighted in the pooling and one participant gave no information. The number of pooled samples in one run was not collected. Finally, sequencing was conducted in most cases with Illumina sequencing devices (11/15) with nine participants utilizing the Illumina MiSeq for sequencing. Two laboratories employed the Thermo Fisher Scientific Ion S5 device and one participant employed beside an Illumina MiSeq a Thermo Fisher Scientific Ion S5 instrument (Table 2).

**Table 2:** Details on the participants’ applied library preparation and sequencing procedures.

Laboratory code	Library Prep Kit	Order no.	Pooling^*^	Sequencing device	Cycles^**^
LC01_LS	Illumina® DNA Prep, (M) Tagmentation (96 Samples)	20018705	weighted	Illumina MiSeq	151 p.e.
LC02_LS	TruSeq Nano DNA Low Throughput Library Prep Kit (24 samples)	20015964	weighted	Illumina MiSeq	251 p.e.
LC03_LS	Illumina® DNA Prep, (M) Tagmentation (24 Samples)	20018704	weighted	Illumina MiSeq	251 p.e.
LC04_LS	Illumina® DNA Prep, (M) Tagmentation (96 Samples)	20018705	weighted	Illumina MiSeq	301 p.e.
LC04_S5_LS	Ion Plus Fragment Library Kit	4471252	weighted	Thermo Fisher Ion S5	850 s.e.
LC05_LS	NEBNext® Ultra™ II FS DNA Library Prep Kit	E7805S	weighted	Illumina NextSeq 2000	151 p.e.
LC06_LS	Nextera XT DNA Library Preparation Kit (96 samples)	FC-131-1096	equally	Illumina NovaSeq 6000	101 p.e.
LC07_LS	NEBNext® Ultra™ II DNA Library Prep Kit	E7645L	weighted	Illumina MiSeq	251 p.e.
LC08_LS	Illumina® DNA Prep, (M) Tagmentation (24 Samples)	20018704	weighted	Illumina iSeq 100	151 p.e.
LC09_LS	Illumina® DNA Prep, (M) Tagmentation (24 Samples)	20018704	equally	Illumina MiSeq	301 p.e.
LC10_LS	Illumina® DNA Prep, (M) Tagmentation (96 Samples)	20018705	weighted	Illumina MiSeq	151 p.e.
LC11_LS	Illumina® DNA Prep, (M) Tagmentation (24 Samples)	20018704	equally	Illumina MiSeq	301 p.e.
LC12_LS	Illumina® DNA Prep, (M) Tagmentation (96 Samples)	20018705	weighted	Illumina MiSeq	251 p.e.
LC13_S5_LS	NEBNext® Fast DNA Library Prep Set for Ion Torrent	E6270L	weighted	Thermo Fisher Ion S5	800 s.e.
LC14_S5_LS	unknown	unknown	unknown	Thermo Fisher Ion S5	unknown
LC01_CE	Illumina® DNA Prep, (M) Tagmentation (96 Samples)	20018705	weighted	Illumina MiSeq	251 p.e.
LC02_CE	TruSeq Nano DNA Low Throughput Library Prep Kit (24 samples)	20015964	weighted	Illumina MiSeq	251 p.e.
LC03_CE	Illumina® DNA Prep, (M) Tagmentation (24 Samples)	20018704	weighted	Illumina MiSeq	151 p.e.
LC04_CE	Illumina® DNA Prep, (M) Tagmentation (96 Samples)	20018705	weighted	Illumina MiSeq	301 p.e.
LC05_CE	NEBNext® Fast DNA Library Prep Set for Ion Torrent	E6270L	weighted	Thermo Fisher Ion S5	850 s.e.
LC06_CE	NEBNext® Ultra™ II FS DNA Library Prep Kit	E7805L	weighted	Illumina NextSeq 2000	150 p.e.
LC07_CE	Nextera XT DNA Library Preparation Kit (96 samples)	FC-131-1096	equally	Illumina NovaSeq 6000	101 p.e.
LC08_CE	NEBNext® Ultra™ II DNA Library Prep Kit	E7645L	weighted	Illumina MiSeq	251 p.e.
LC09_CE	Nextera XT DNA Library Preparation Kit (96 samples)	FC-131-1096	weighted	Illumina MiSeq	300 p.e.
LC10_CE	Illumina® DNA Prep, (M) Tagmentation (96 Samples)	20018705	weighted	Illumina MiSeq	151 p.e.
LC11_CE	NEBNext® Fast DNA Library Prep Set for Ion Torrent	E6270L	weighted	Thermo Fisher Ion S5	unknown
LC12_CE	Illumina® DNA Prep, (M) Tagmentation (96 Samples)	20018705	equally	Illumina MiSeq	301 p.e.
LC13_CE	Illumina® DNA Prep, (M) Tagmentation (24 Samples)	20018704	weighted	Illumina iSeq 100	151 p.e.
LC14_CE	Nextera XT DNA Library Preparation Kit (96 samples)	FC-131-1096	equally	Illumina MiSeq	unknown
LC15_CE	Illumina® DNA Prep, (M) Tagmentation (96 Samples)	20018705	weighted	Illumina MiSeq	201 p.e.

* Pooling was performed either weighted according to the genome size or equally (every sample the same amount of library DNA).

** p.e. = paired-end, s.e. = single-end.

In the ring trial part II, six different kits were used to prepare the libraries for sequencing. Among them, 7/15 participants used the Illumina® DNA Prep, 3/15 participants used the Nextera XT DNA Library Prep Kit, and the two participants with an Ion S5 sequencer used the NEBNext® Fast DNA Library Prep Set for Ion Torrent. Some participants reported deviations from the kit protocol, e.g., halving the reagents’ volumes (4 participants, Illumina® DNA Prep and Nextera XT DNA Library Preparation Kit), use of 70% instead of 80% (v/v) ethanol, usage of different beads and different incubation time (Supplementary Table 1). According to the kits’ protocol, DNA was enzymatically fragmented in 11 cases and mechanically in 4 cases. When pooling the libraries, 12 participants weighted them according to the genome size and three participants (LC07, LC12, and LC14) included all probes equally weighted in the pooling while the overall number of samples sequenced in one run was not collected. For sequencing, the majority of institutions (13/15) applied Illumina sequencing devices, thereof 10 participants employing the Illumina MiSeq for sequencing. Two institutions employed the Thermo Fisher Scientific Ion S5. In both ring trial parts, the number of cycles used in paired-end sequencing varied from 2× 101 to 2× 301 cycles.

As the ring trials only concerned the quality of the sequencing data, participants were not required to conduct further analyses. Nevertheless, when asked, which software they would usually use for genome assembly and quality control, a number of different tools were listed. In the ring trial part I, participants would use the AQUAMIS pipeline (6x, https://gitlab.com/bfr_bioinformatics/AQUAMIS), Ridom SeqSphere+ (1x, https://www.ridom.de/seqsphere/), CLC Workbench (1x, https://digitalinsights.qiagen.com/products-overview/discovery-insights-portfolio/analysis-and-visualization/qiagen-clc-genomics-workbench/) and a list of software including for assembly Shovill (1x, https://github.com/tseemann/shovill) or SKESA (2x, https://github.com/ncbi/SKESA) or SPAdes (2x, https://github.com/ablab/spades). Two participants provided no information. In the ring trial part II, participants would use the AQUAMIS pipeline (8x), Ridom SeqSphere+ (1x) and several software programs including Shovill (1x) or SKESA (1x) or SPAdes (1x) for assembly. Three participants provided no information. The corresponding software versions can be found in Supplementary Table 1.

### 3.2 Quality assessment based on Q30 base fraction, contamination control, assembly length and coverage, N50 and full genes

**Figure 3:**
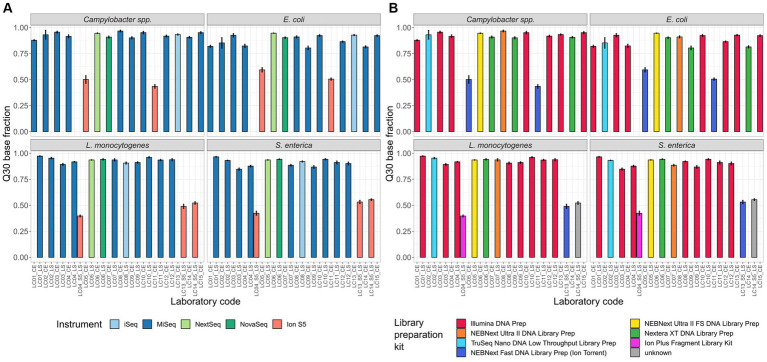
Percentage of bases with a Phred quality score ≥ 30. Depicted is the mean of the Q30 base fraction including all isolates per participant, coloured by **(A)** the employed sequencing device and **(B)** the employed library preparation kit.

Q30: We found meaningful variability in the Q30 base fraction (after trimming) between the sequencing data provided by the Illumina and Thermo Fisher Scientific (Ion Torrent) instruments independent of the species. The mean of the Ion S5 Q30 base fraction for all samples of all four species was nearly half compared to the mean Q30 base fraction of Illumina sequencing platforms (Illumina instruments 91.3% [547 samples] vs. Ion S5 49.6% [110 samples]; Figure 3). When comparing the Q30 base fraction of sequence data derived from sequencing devices of the same manufacturer values were more homogenously. There was little variability between the mean of different Illumina devices: iSeq 92.2% [43 samples, SD 1.2%], MiSeq 90.8% [416 samples, SD 4.6%], NextSeq 94.1% [44 samples, SD 0.5%], NovaSeq 92.4% [44 samples, SD 1.9%] (Figure 3). Additionally, we affirmed that the Q30 base fraction is negatively correlated with the number of sequencing cycles. Notably, participants applying 301 cycle paired-end (p.e.) sequencing run did not always achieve a Q30 base fraction of over 80%, while participants with less cycles did. Regarding the different Illumina library preparation kits, as well as the four structurally different species, no obvious differences in the Q30 base fraction were observed (Figure 3).

Contamination: Overall, the data of six samples was found to be strongly contaminated. Two samples of participant LC02_LS (LC02-21-RV3-P64-S03 and LC02-21-RV3-P64-L09) were intra-genus contaminated with >30 contaminating single nucleotide variants. Additionally, the bacterial genome assembly failed. Furthermore, the data of sample LC14-22-RV4-P64-C04 had 113 contaminating single nucleotide variants and two MLST loci with multiple alleles. Three samples of participant LC15_CE (LC15-22-RV4-P64-C02, -C06 and -C09) were found to be inter-genus contaminated, with more than 5% of reads on read-level and 5% of contigs on contig-level belonging to a different genus. In detail, for sample LC15-22-RV4-P64-C02/-C06/-C09 6/25/10% of reads and 7/38/56% of contigs, respectively, were from a different genus (e.g., *Staphylococcus*, *Neisseria*, *Streptococcus*). The AQUAMIS pipeline sensitively detected more samples with only minor contaminations, however, these contaminations were probably due to low scale index bleeding or between-run carry over and were not considered disruptive for further analysis as cgMLST or SNP analysis.

**Figure 4:**
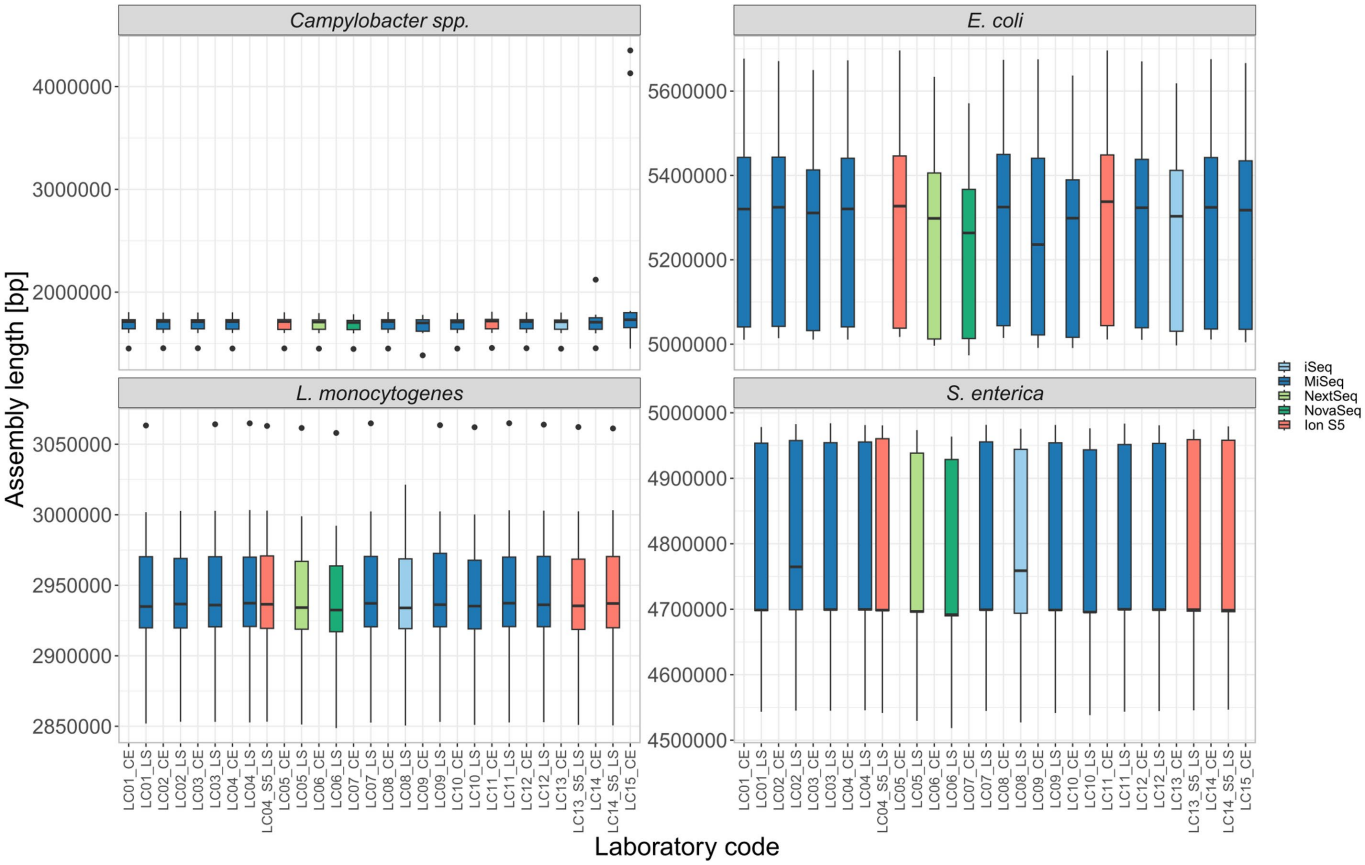
The assembly length of all isolates per participant.

Assembly length: The mean total assembly length for all isolates per different participant was overall comparable (Figure 4). Outliers with a significantly longer assembly were found for the contaminated *Campylobacter* spp. samples LC14-22-RV4-P64-C04 (intra-genus), LC15-22-RV4-P64-C06 and LC15-22-RV4-P64-C09 (both inter-genus contaminated). Additionally, the inter-genus contamination of LC15-22-RV4-P64-C02 lead to a ~ 100.000 bp longer assembly, thereby altering the median assembly length of LC15_CE in comparison to other participants (Figure 4). The increased median of LC02_LS and LC08_LS for *S. enterica* (Figure 4) can be explained by the missing samples in the calculation for the median.

Assembly coverage depth: The assembly coverage depth varied per participant, isolate and species (Figure 1 in Supplementary Data Sheet 1). The median coverage depth for all samples of all participants per species was 92.2 (interquartile range (IQR) 22.7) for *Campylobacter* spp., 69 (IQR 24.7) for *E. coli*, 80.8 (IQR 39.2) for *L. monocytogenes* and 81.1 (IQR 32.3) for *S. enterica,* based on 163 to 165 samples per species/genus. As described earlier, the pooling approach was open to the participants (pooling according to genome size or every sample equally). To enforce reliable assemblies of good quality, participants were asked to provide enough data for an assembly coverage depth of minimum 20x (40x for *E. coli* and 30x for *S. enterica*). This requirement was met for all *Campylobacter* spp. samples, *L. monocytogenes* and for *S. enterica* samples (minimum 23.1, 22.8 and 32.0, respectively). For *E. coli* samples the minimum assembly coverage depth was not obtained for seven samples of three participants (LC03_CE 2x, LC05_CE 1x, LC13_CE 4x, minimum 32).

**Figure 5:**
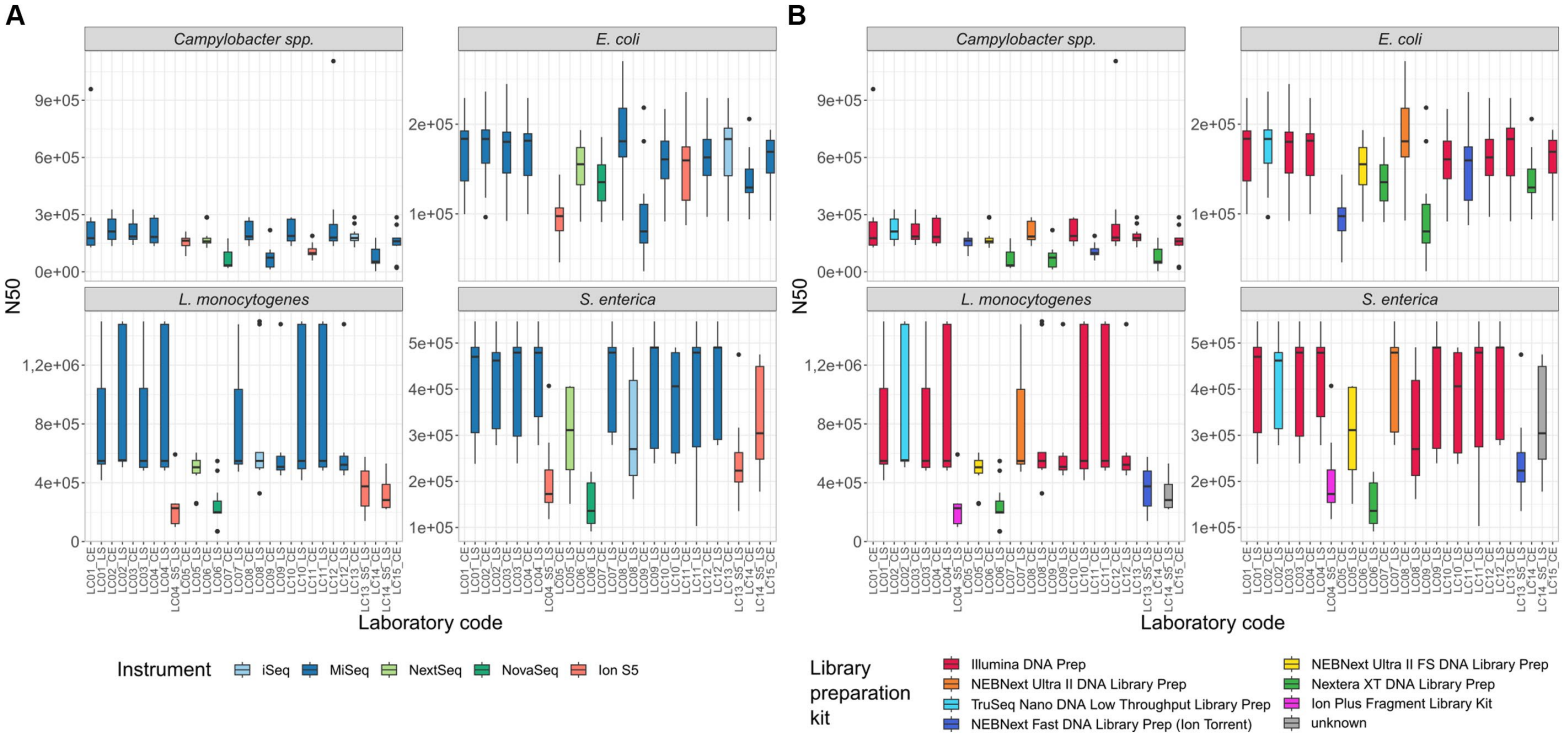
N50 of all isolates per participant. **(A)** Coloured by sequencing instrument, **(B)** coloured by library preparation kit.

N50 and full genes: N50 values differed in parts highly between the samples of different participants (Figure 5). For the *Campylobacter* spp. assemblies, the lowest N50 values were calculated with participants applying the Nextera XT DNA Library Prep kit (median N50 LC07_CE (NovaSeq) 34,262; LC09_CE (MiSeq) 74,552; LC14_CE (MiSeq) 53,284 vs. median N50 *Campylobacter* spp. isolates overall 163,373). With the *E. coli* assemblies, the same participants had again the lowest N50 values in addition to one Ion S5 user (median N50 LC05_CE (Ion S5) 97,516; LC07_CE (NovaSeq) 135,375; LC09_CE 80,470 (MiSeq); LC14_CE 129,396 (MiSeq) vs. median N50 *E. coli* isolates overall 155,468). With the *L. monocytogenes* and *S. enterica* isolates, the lowest median N50 values were again observed with Ion S5 instruments in combination with different library preparation kits, as well as the only participant applying the Nextera XT DNA Library Prep kit (in combination with a NovaSeq). Samples prepared with the NEBNext Ultra II DNA Library Prep kit, the Illumina DNA Prep kit and the TruSeq Nano DNA Low Throughput kit had comparably good N50 values. Overall, for three out of four species investigated, the Nextera XT DNA Library Prep kit prepared samples had the lowest median N50 value after assembly (Supplementary Table 2). Taking into consideration only the library preparation kits for Illumina sequencing devices, it was four out of four species investigated. Likewise, the median number of full genes is reduced in samples prepared with Nextera XT DNA Library Prep kit in comparison with, e.g., the Illumina DNA Prep kit (Figure 2 in Supplementary Data Sheet 1).

Details on the median of the quality characteristics Q30, assembly length, coverage depth, N50 and number of full genes, grouped by species, instrument and sequencing platform, can be found in the Supplementary Table 2.

### 3.3 Principal component analysis

**Figure 6:**
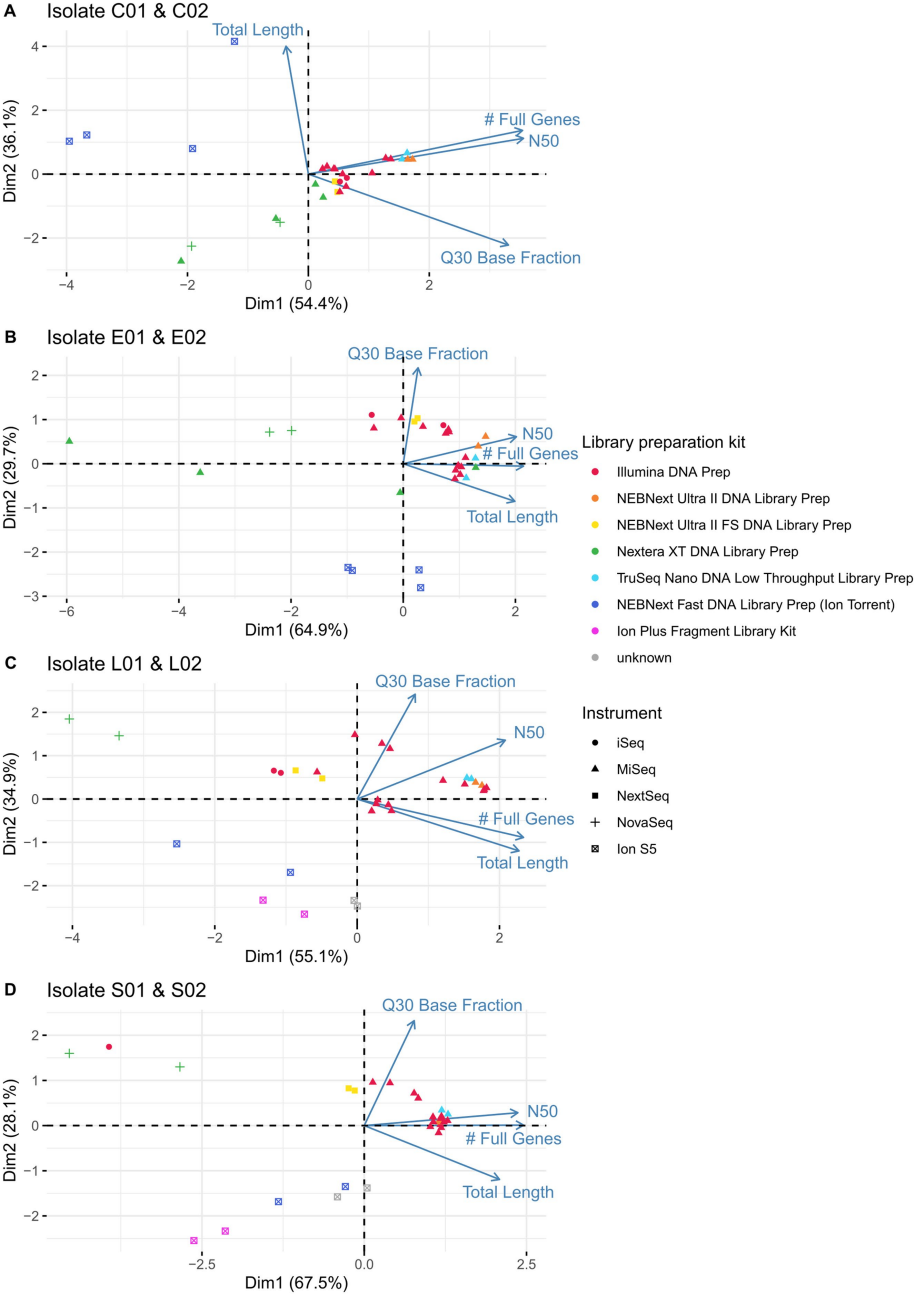
Principal component analysis on the identical isolates of **(A)** *Campylobacter jejuni*, **(B)** *Escherichia coli*, **(C)** *Listeria monocytogenes* and **(D)** *Salmonella enterica* processed by different participants taking into account the variables of Q30 base fraction, N50, number of full genes and total assembly length. Applied library preparation kits are represented by different colours, sequencing instruments by different shapes.

The previous paragraph describes important measures of sequence quality in detail. However, looking at each quality score individually does not permit a full picture, as different parameters may combine to shape the overall quality. To gain a deeper understanding of these combined effects, especially, differences between applied kits and sequencing instruments, we performed a principal component analysis (PCA). As the isolates themselves are very different in nature, only the duplicate isolates of the ring trials (C01 and C02, E01 and E02, L01 and L02, S01 and S02) were analysed in corresponding PCAs. Dubious samples were excluded (LC03-22-RV4-P64-C02 - contaminated; LC15-22-RV4-P64-C02 - interchanged) and no data was available for LC08-21-RV3-P64-S01 due to problems in the library preparation. We found, that the data derived from samples prepared with the Illumina DNA Prep kit and sequenced on Illumina MiSeq (red triangels) clustered together (Figure 6). These are similar to data from participants applying the TruSeq Nano DNA Low Troughput kit (light blue triangels) and NEBNext Ultra II DNA Library Prep (orange triangels) in combination with the Illumina MiSeq instrument, as well as sequence data from the NEBNext Ultra II FS Library Preparation kit in combination with the Illumina NextSeq device (yellow squares). Located far away from these clusters are the sequence data prepared with NEBNext Fast DNA Library Prep for Ion Torrent or Ion Plus Fragment Library Kit and sequenced with Ion S5 due to lower Q30 and N50 values. Additionally, the sequence data prepared with the Nextera XT DNA Library Preparation kit clusters separate to data derived from other library preparation kits (green triangels and crosses), independent of the applied sequencing device (Illumina Miseq/ NovaSeq), having a lower number of full genes, lower N50 value and assembly length. These findings hold true for the analyzed samples of the four investigated species.

### 3.4 Determination of 7-gene multilocus sequence type

The 7-gene MLST sequence type was correctly determined for all *E. coli* and *L. monocytogenes* assemblies. For five *Campylobacter* spp. assemblies of three participants no sequence type could be assigned, as well as for one *S. enterica* assembly (Supplementary Table 3, including contaminated samples). In most cases, only one allele was misclassified, in one case different alleles were found for the same loci (Supplementary Table 3). Reasons for incorrect sequence types were attributed to aforementioned intra-genus or inter-genus contamination or fragmented genomes. Additionally, four *Campylobacter* samples of one participant were mixed-up, with the assemblies showing correct sequence types for the swapped samples (Supplementary Table 3).

### 3.5 Analysis of cgMLST calling

**Figure 7:**
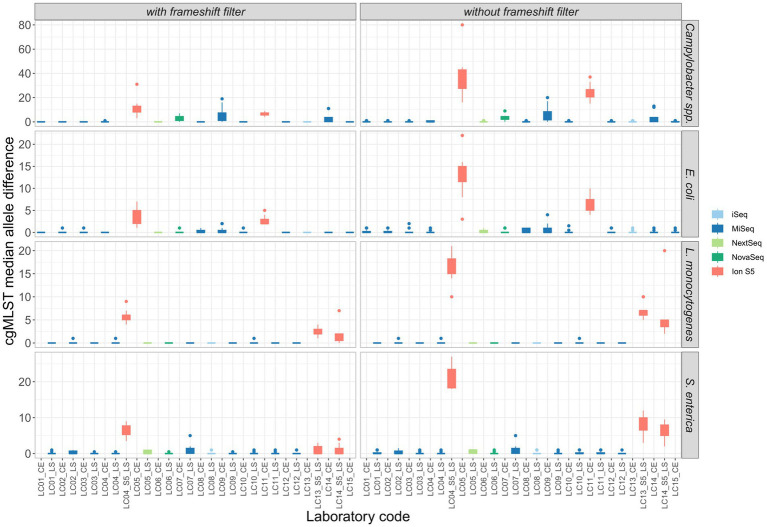
Comparison of the median allele difference (of one assembly to other assemblies within identical isolates) per participating laboratory and the effect of a frameshift filter (>5% deviation from the median length) on the median of cgMLST allele differences of identical isolates sequenced by different participants.

To investigate the impact of variability on clustering, cgMLST analyses were performed on the assemblies of the participants’ data for the four species with exclusion of strongly contaminated samples. All cgMLST distance matrices are presented in Supplementary Table 4. For easy comparison, the median cgMLST distance was calculated from the allelic differences of one assembly to other assemblies within identical isolates (see also Uelze et al., 2020a). Assemblies derived from Ion Torrent short read data generally displayed a much higher number of allele differences compared to those constructed from Illumina short reads (Figure 7). The allele difference was reduced with the use of a frameshift filter, which neglects alleles that deviate more than 5% of the median length of that allele (Figure 7). Application of the frameshift filter had a proportionally greater effect on allele differences of Ion Torrent derived assemblies. Within the assemblies based on Illumina data, the highest median allele difference was observed for the assemblies based on data from participants of LC07_CE, LC09_CE, and LC14_CE in case of *Campylobacter* and *E. coli* isolates, all of them applying the Nextera XT DNA Library Prep Kit. The observation is in congruence with a higher number of contigs and a lower N50 value, as described above, and the effect was found to be stronger for the *Campylobacter* spp. isolates.

**Figure 8:**
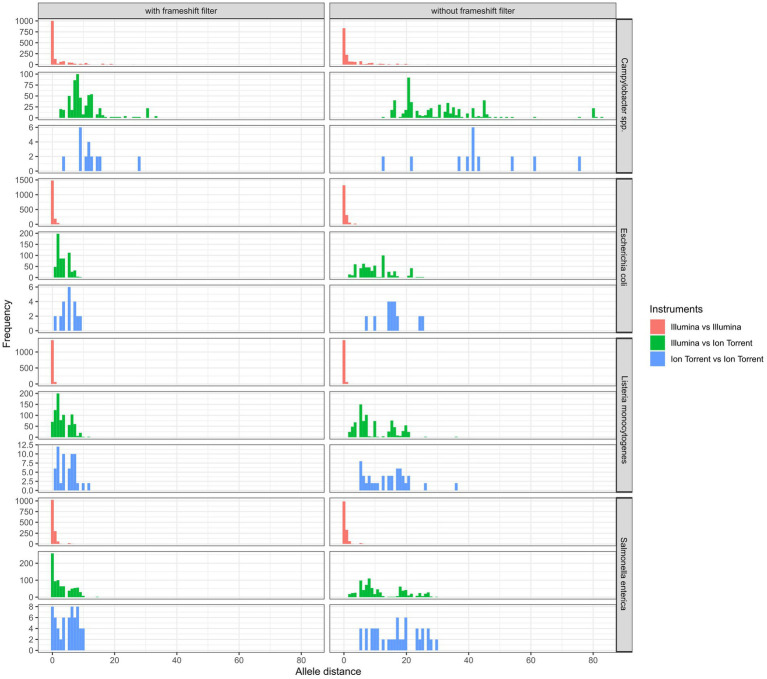
Comparison of cgMLST allele distances (of one assembly to other assemblies within identical isolates) between same and different sequencing technologies with and without the application of a frameshift filter in the analysis.

In general, the lowest amount of allele differences was observed within assemblies based on Illumina data, followed by the Ion Torrent-Illumina and Ion Torrent-Ion Torrent pairs, respectively (Figure 8). It should be noted that the number of value pairs for the Illumina-Illumina assembly comparison is higher, than for other combinations, since the majority of participants employed Illumina devices in the ring trial. The allelic distances between assemblies per isolate are highest for *Campylobacter* spp. assemblies, with 4.2 allele distances in the mean (with frameshift filter applied), compared to 1.0, 1.2, and 1.3 for *E. coli*, *L. monocytogenes* and *S. enterica* assemblies, respectively. The high number of allele differences in case of *Campylobacter* spp. correspond to the assemblies of two Ion Torrent data sets and three Nextera XT-based Illumina data sets. When taking these out of the comparison, the allelic distance between the isolates of the remaining 10 participants (applying 4 different library prep kits) is max. 1, with a mean of 0.1 to 0.4 (with frameshift filter applied: 0.01 to 0.8). The effect is similar for the other species.

### 3.6 Analysis of SNP calling

**Figure 9:**
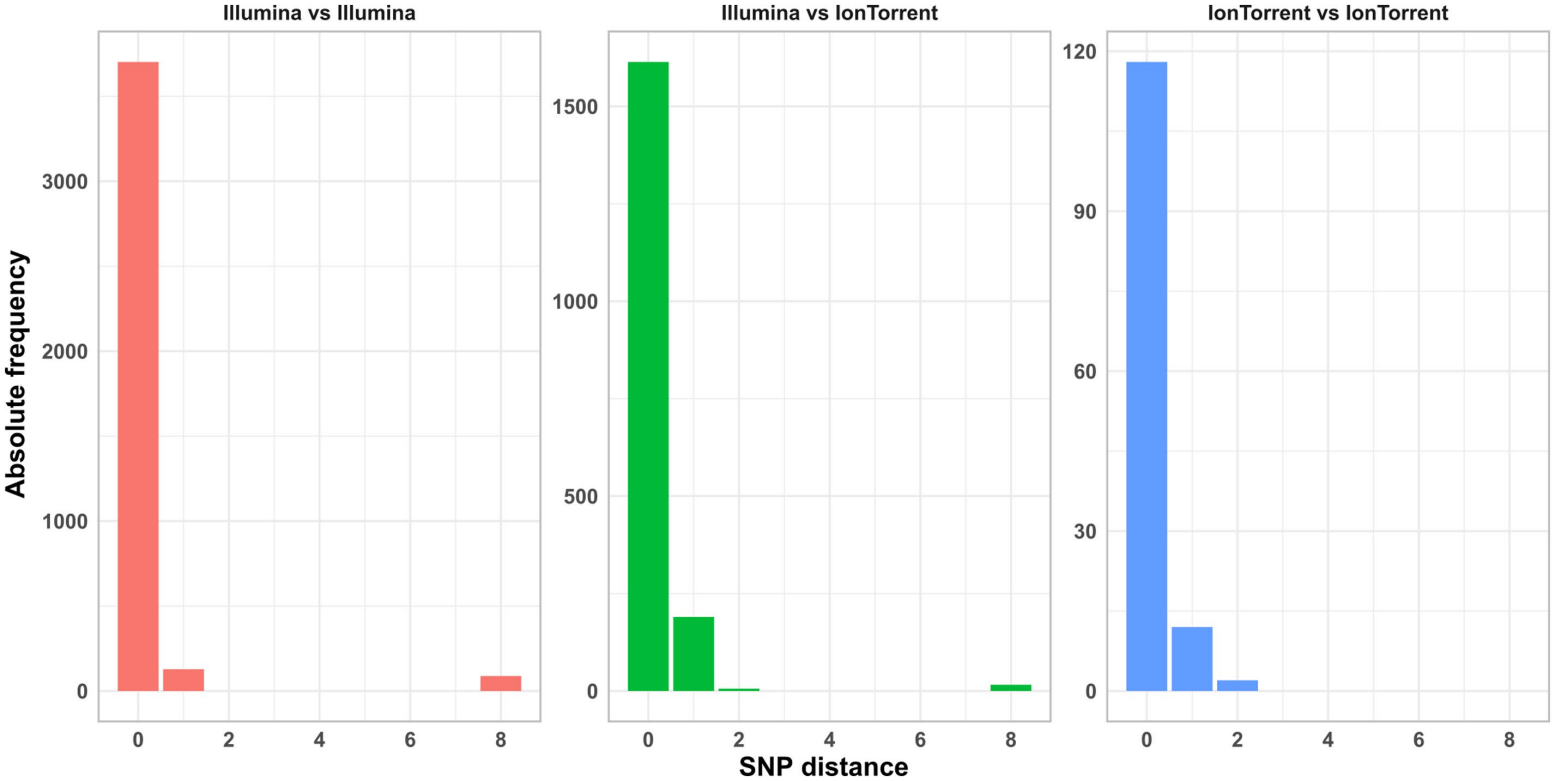
Absolute frequency of SNP distances in the sequence data of different sequencing device manufacturers.

**Figure 10:**
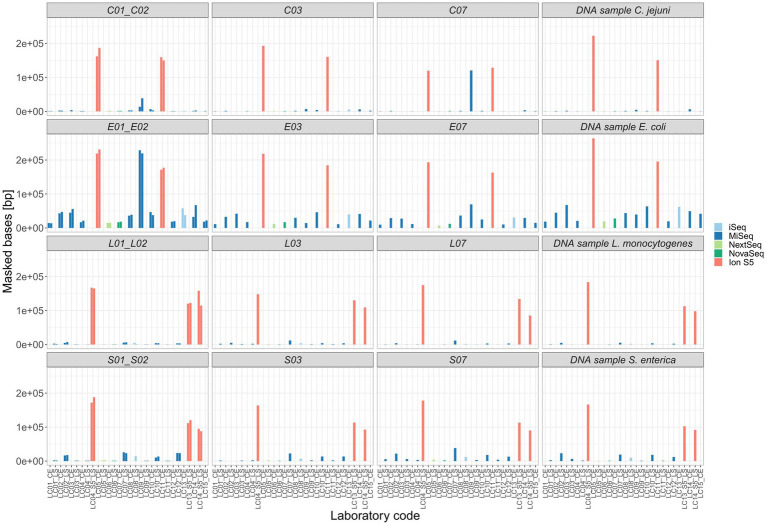
Masked bases unconsidered in the core-alignment of each SNP analysis. Selected ring trial isolates were analyzed in individual SNP analyses with exception of the duplicated isolate (X01_X02) that was analyzed in one SNP analysis.

For five isolates per species (including the duplicated isolate and the DNA control) individual SNP analyses were performed. Strongly contaminated samples were excluded. All SNP distance matrices are provided in Supplementary Table 5. Overall, only a small number of SNPs were detected in the sequence data independent of the application of specific sequencing devices/manufacturers (Figure 9). SNPs occurred exclusively in cultured samples, not in the DNA controls (Figure 3 in Supplementary Data Sheet 1). However, the number of masked bases in the analysis was more than tenfold for Ion S5 data compared to Illumina data (Ion S5 mean: 151,150 bases [50 samples] vs. Illumina mean: 13,423 bases [244 samples]), therefore reducing the size of the core genome (Figure 10). The number of uncovered bases remained roughly the same independent of the applied sequencing device (Figure 4 in Supplementary Data Sheet 1).

### 3.7 Analysis of the assembler effect: SPAdes vs. SKESA

To determine if the two assemblers SPAdes and SKESA differentiate in their suitability for the assembly of data of different species, library preparation and/or sequencing methods, thereby impacting the assessed quality characteristics, the assembling step was repeated applying SKESA instead of SPAdes (both through shovill in AQUAMIS). Applying SPAdes, the mean assembly metrics showed slightly longer assemblies, higher N50 values and a higher number of full and partial genes in the assemblies of the isolates of all investigated species (Table 3). However, the differences were small. When comparing specific library preparation kits combined with different sequencing devices in detail, the results were supporting the general trend: In the vast majority the SPAdes and SKESA assemblies QC metrics were comparable, sometimes slightly higher/better for SPAdes assemblies (Supplementary Table 6). This also holds true for the number of full and partial genes detected. However, there was one exception: In the case of the *Campylobacter* spp., *L. monocytogenes*, and *S. enterica* isolates in combination with a NovaSeq device and the Nextera XT DNA Library Prep, more full genes were detectable in the SKESA assemblies. Even though, other combinations of sequencing instruments and library preparation kit achieved a greater number of detected genes for these isolates regardless the assembler. For example, the combination of Illumina MiSeq (instead of NovaSeq) and Nextera XT DNA Library Prep followed the general trend in SPAdes delivering minimal better assemblies.

**Table 3:** Mean characteristics of ring trial’s Illumina data of the four food-associated pathogens assembled with SKESA or SPAdes.

Species	Assembly	*n*	Mean coverage	Mean length	Mean contigs >1,000 bp	Mean N50	Mean full genes	Mean partial genes
*Campylobacter* spp.	SKESA	139	88.2	1689311.0	45.1	159042.4	1677.8	73.8
	SPAdes	139	87.2	1717220.0	38.3	180156.0	1699.8	63.6
*E. coli*	SKESA	143	70.5	5258797.5	130.9	142857.4	4915.1	141.8
	SPAdes	143	70.1	5284852.5	116.6	156652.3	4949.7	141.9
*L. monocytogenes*	SKESA	131	78.9	2942729.7	17.3	628120.3	3021.6	21.0
	SPAdes	131	78.9	2946314.9	15.0	707883.7	3036.7	16.1
*S. enterica*	SKESA	130	76.6	4781988.5	39.9	342785.7	4750.8	38.8
	SPAdes	130	76.6	4791453.0	36.5	366104.6	4765.5	45.8

*n*: number of data sets.

**Figure 11:**
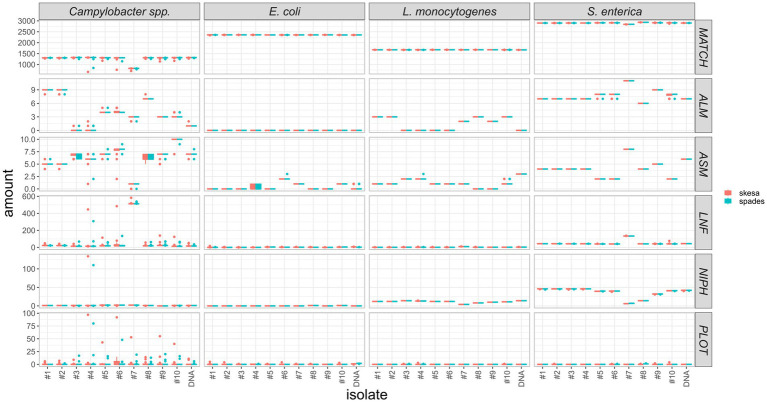
Comparison of cgMLST statistics of assemblies created with SPAdes or SKESA software. Y-axis represents number of MATCH = exact allele matches and inferred new alleles, ALM/ASM = number of alleles 20% larger/shorter than length mode of the distribution of the matched loci, LNF = number of loci not found, NIPH = number of non-informative paralogous hit, PLOT = possible loci on the tip of the query genome contigs, respectively.

As assemblies are the input for cgMLST analysis we also compared allele calling statistics for the two assemblers, such as the sum of exact allele matches and inferred new alleles (MATCH), number of alleles 20% larger or shorter than length mode of the distribution of the matched loci (ALM/ASM), number of loci not found (LNF), number of non-informative paralogous hits (NIPH), and possible loci on the tip of the query genome contigs (PLOT). We found, that the results are overall highly comparable with few outliers (Figure 11; Supplementary Table 7). They indicate an equivalent aptitude of SPAdes and SKESA for genome assembly of *E. coli*, *L. monocytogenes*, and *S. enterica* Illumina data for subsequent cgMLST analysis. For *Campylobacter* spp., the results are slightly more deviating, however, there is no clear trend (Figure 11).

## 4 Discussion

In a survey of 2015, on behalf of the Global Microbial Identifier initiative, participants were asked to delineate priority pathogens for inclusion in a proficiency testing that will look at all stages of sequencing and analysis. The first five with the highest consent comprised *Salmonella* spp., *E. coli*, *Campylobacter* spp., *S. aureus*, and *Listeria* spp. (Moran-Gilad et al., 2015). In line with this, we investigated the effects of different library preparation and sequencing methods on data quality of bacterial whole genome sequencing and the implications on cluster analysis, involving the four species accounting for the majority of food infections: *E. coli*, *L. monocytogenes*, *S. enterica*, and *Campylobacter* spp. Other large published ring trials based on foodborne pathogens and short-read sequencing prescribed a certain laboratory procedure for library preparation and sequencing, like the GenomeTrakr proficiency testings of 2015 to 2018 (Timme et al., 2018, 2020a). In this standardized approach, laboratories achieve highly comparable data with only minimal differences. Other studies have been published, which allowed laboratories to choose their own library preparation or sequencing methods (Pasquali et al., 2019; Dylus et al., 2020; Lau et al., 2021), mostly focusing on a few samples of one species. To our knowledge, this ring trial is unique in the number of species and isolates analyzed while encouraging the participants to follow their routine protocols. Laboratory protocols were highly variable, comprising a broad range of DNA extraction kits, library preparation kits and sequencing instruments.

Overall, 659 raw data sets were analyzed, comprising 10 isolates and one DNA control of four species each sequenced by 14 to 15 participants. This large and diverse dataset contains valuable information and can be used to investigate the effects of different sequencing routines on data quality and downstream analyses, e.g., comparison of sequencing technologies or library preparation kits, their suitability for the tested species, and the performance of individual laboratories. In this publication, we focused on the comparability of data and the possible reasons for variability. As the raw sequence data and the corresponding metadata provided by the participants is openly accessible, we encourage other scientists to use this data for their own studies, as not every aspect can be covered in detail in this publication.

Generally, we found, that the participants’ sequence data were of high quality. Exceptions could be grouped into three categories: We observed considerable differences in the raw sequence qualities and subsequent assembly qualities that were attributed to

- the difference of Illumina and Ion Torrent sequencing technology. Inherent to the Ion Torrent semiconductor technology are lower base calling qualities in comparison to the optical-based Illumina technology (Loman et al., 2012). In congruence, lower N50 values, lower amount of full genes and higher number of allele differences were found in the assemblies, independent of the species analyzed, when compared to Illumina datasets. This finding was also unaffected by the choice of the applied library preparation kit and equally observed in the DNA control. Interestingly, SNP analysis results showed no sequencing technology-specific difference, however, a higher proportion of bases was masked in the core-alignment, when including Ion Torrent data. Masked bases affect the size of the core-genome on which SNPs are called and therefore diminish the resolution in subsequent analyses (e.g., clustering).
- choice of the Illumina library preparation kit. Regarding the Illumina library preparation kits, raw data of four from five kits achieved similar values when comparing Q30 percentage, N50 values, number of full genes, allele differences and number of SNPs. Sequence data produced with the fifth kit (Nextera XT library preparation kit) on the other hand was of significantly lower quality. While the Q30 values were comparable, N50 values were lowered and as a direct consequence, fewer full genes could be detected. This effect was strongest for *Campylobacter* spp. but also apparent for *E. coli*. The same effect was observed in the first part of the ring trial comprising *L. monocytogenes* and *S. enterica* samples, where the only participant applying the Nextera XT kit obtained assemblies with significantly lower N50 values. This is not a new finding, and other previous studies have conclusively shown that the Nextera XT preparation is affected by GC content variability within and between genomes (Seth-Smith et al., 2019), believed to be connected to G and C residues in the tagmentation motif (Segerman et al., 2022). Thus species with overall low GC-content, such as *Campylobacter* spp. (which has the lowest GC content of the four species under investigation) are more likely to be affected by the GC bias of the library preparation kit. GC biased sequence data can affect subsequent analyses such as assignment of allele differences or serovar detection (Uelze et al., 2020b). We would like to encourage researchers to consider and investigate the GC bias of their library preparation kits and avoid the use of less suitable kits.
- contaminated samples. Overall, six samples were found to be contaminated with the same or another species. Additional four samples were simply swapped. Contaminated samples were identified during contamination control in the initial quality check and showed in some cases longer assembly lengths, as well as deviating GC contents. As contaminated and swapped samples would have skewed cgMLST and SNP analysis results, they were excluded from further analyses. Independent of the kit and instrument chosen for library preparation and sequencing, careful sample handling is of utmost importance to prevent any kind of contamination and mix-up.

Due to the broad range of applied DNA extraction kits, more than one DNA sample would be required to reliably assess variability introduced by different DNA extraction kits. However, the combined effects of different library preparation kits and sequencing instruments are likely to overshadow the effects of different DNA extraction kits. No core SNPs were detected in the data of the species’ DNA samples, as well as in the majority of cultured isolate data, indicating that the culturing is a minor source of variability.

When evaluating which combinations of library preparation kit and sequencing technology have an effect on sequence data quality, we found that results of a principal component analysis generally supported the above findings of the individual quality metrics. Calculated on the quality metrics of the duplicates of each species, lllumina sequenced samples clustered closely together, with the Nextera XT prepared libraries located more distantly. The in comparison fewer samples sequenced with Ion Torrent instruments cluster together, however, less dense and apart from Illumina sequenced samples. Reproducibility of the two identical isolates on laboratory-level is not presented (with exception of the PCA analysis) as we focused on the description of major differences in the sequencing data between laboratories and their differing wet-lab approaches. Sufficient sequence data quality is of course not an end in itself, but highly relevant for any subsequent analyses in respect to the correctness, accuracy and uncertainty of the result. For example, the sequence type could not be assessed correctly for all samples in this study. Wrong sequence types affected mostly *Campylobacter* spp. assemblies, as well as one *S. enterica* assembly. A wide range of possible causes was identified, such as interchanged samples, contamination, fragmented genomes of Ion Torrent sequenced samples and samples prepared with the Nextera XT kit.

In the cgMLST analysis, the highest number of allele differences was identified for *Campylobacter* spp., followed by *S. enterica, L. monocytogenes*, and *E. coli*. The allele difference between Illumina-based assemblies was consistently smaller, compared to the combination of Ion Torrent–Illumina and in-between Ion Torrent assemblies, independent of the species analyzed. The application of a frameshift filter, which removes alleles that deviate more than 5% of the median length of that allele, significantly reduced the allele differences of the combination of Ion Torrent–Illumina and in-between Ion Torrent assemblies. However, even with this filtering step, a larger number of allele differences were detected for Ion Torrent-based assemblies, compared to Illumina-based assemblies. In a common cluster analysis, e.g., for the analysis of an outbreak, the so caused allelic distance could lead to a false exclusion of involved isolates. In contrast, SNP analysis revealed no relationship between the type of sequencing technology and the number of SNPs. While this finding is favorable for cluster analysis and outbreak detection, it does not indicate that all data types are equally. Instead, the seemingly homogeneity of the sequence data in SNP calling is achieved through yet another filtering mechanism which is implemented and routinely performed in the Snippy software, as variants with heterozygous or poor quality genotype are not considered as true variants. By calculation of the amount of thereby masked positions, we found that the number of masked bases was tenfold higher in Ion Torrent datasets. In the context of a centralized data analysis, it would be optimal to share quality-checked sequencing data in combination with metadata on sample preparation (e.g., applied library preparation kit and sequencing technology). The amount of unaligned bases showed no influence by the applied technology. The amount at which each data set contributes to a reduction of the core genome is an important factor as it reduces resolution and sensitivity of the overall SNP analyses.

Besides the investigated effect of wet-lab procedures on variability in the data analysis, laboratories choosing sequencing technology should also consider the availability and quality of bioinformatics software for their data types. For example, while the here applied assembly pipeline does accept Ion Torrent data as input, it was optimized for Illumina data. This reflects a general trend that is a consequence of the market share of Illumina on high-throughput instruments, which is estimated to be over 90%. Thus, most open source software tools are developed for Illumina data as main input source. We further compared the performance of SKESA and SPAdes on the data sets generated with Illumina devices and found only minimal differences in the assembly quality, with SPAdes slightly ahead and equivalent results in cgMLST statistics. In a unique combination of Nextera XT library preparation kit and NovaSeq sequencing, a higher number of full genes were detected in the SKESA assemblies, showing that the definition of the “best” assembler is dependent on the data and its intrinsic characteristics. We have excluded Ion Torrent data sets for the comparison because SKESA is not designed for Ion Torrent data.

Finally, as the implementation of a standardized protocol across laboratories is not feasible in Germany, it is crucial to identify factors within wet-lab protocols contributing to low sequence quality and to minimize their negative impact. In this study, we could attribute lower sequence quality at the library preparation level to the Nextera XT library preparation kit as well as generating sequences using the Ion Torrent device S5. Undesired effects, caused by the inclusion of Ion Torrent data in cgMLST or SNP analysis can be mitigated through the use of frameshift-filter in the cgMLST analyses or the masking of bases in the SNP analysis. However, filtering requires the definition of an adequate cut-off to exclude technical noise but retain sensible information, without loss of sensitivity. Therefore, filters should be applied only when appropriate. In our study, with the exception of the Nextera XT kits, all applied Illumina kits and Illumina sequencing devices worked comparably well. This study focused on the variability introduced by the wet-lab procedures necessary for generating the data, while maintaining a central data analysis. Of course, analyses results are also affected by the choice and parameter-settings of different bioinformatics tools. To address this aspect, we plan to conduct an interlaboratory study, focused on the bioinformatics analysis and cluster evaluation of the four species targeted in this study.

## Data availability statement

The sequence data generated in this study has been deposited in the publicly available online repository European Nucleotide Archive (ENA) with the BioProject number PRJEB62505. Detailed original result reports of the ring trials in German language can be assessed at the website of the German Federal Office of Consumer Protection and Food Safety (Forth 2022; Uelze 2021).The code for the AQUAMIS, ChewieSnake and SnippySnake bioinformatic pipelines is freely available from GitLab (https://gitlab.com/bfr_bioinformatics/).

## Author contributions

LF: Conceptualization, Data curation, Writing – review & editing, Formal analysis, Investigation, Methodology, Visualization, Writing – original draft. EB: Investigation, Writing – review & editing. GD: Investigation, Writing – review & editing. AF: Investigation, Writing – review & editing. SF: Investigation, Writing – review & editing. JaF: Investigation, Writing – review & editing. A-CG: Investigation, Writing – review & editing. TH: Investigation, Writing – review & editing. EH: Investigation, Writing – review & editing. LM: Investigation, Writing – review & editing. HP: Investigation, Writing – review & editing. RR: Investigation, Writing – review & editing. ChS: Investigation, Writing – review & editing. ClS: Investigation, Writing – review & editing. KaS: Writing – review & editing, Funding acquisition, Project administration. AT: Investigation, Writing – review & editing. AW: Investigation, Writing – review & editing. JeF: Investigation, Writing – review & editing. SL: Investigation, Writing – review & editing. MP: Investigation, Writing – review & editing. KeS: Investigation, Writing – review & editing. MB: Investigation, Writing – review & editing, Conceptualization, Methodology, Writing – original draft. CD: Writing – review & editing, Formal Analysis, Software. BM: Conceptualization, Writing – review & editing, Methodology, Project administration, Supervision, Writing – original draft. LU: Conceptualization, Methodology, Supervision, Writing – original draft, Writing – review & editing, Data curation, Visualization.

## Supplementary material

The Supplementary material for this article can be found online at: https://www.frontiersin.org/articles/10.3389/fmicb.2023.1253362/full#supplementary-material

Supplementary Data Sheet 1 Supplementary figures 1-4.

Supplementary Table 1 Metadata regarding sample preparation by the participants.

Supplementary Table 2 Summarized quality metrics grouped by species, sequencing device and library preparation kit.

Supplementary Table 3 Cases of deviating 7-gene MLST sequence type.

Supplementary Table 4 cgMLST allele distance matrices.

Supplementary Table 5 SNP distance matrices.

Supplementary Table 6 Detailed mean quality metrics on the genomes assembled with SPAdes and SKESA including investigated species, library preparation kit and sequencing instrument.

Supplementary Table 7 CgMLST statistics of assemblies created with SPAdes or SKESA software. MATCH = exact allele matches and inferred new alleles, ALM/ASM = number of alleles 20% larger/shorter than length mode of the distribution of the matched loci, LNF = number of loci not found, NIPH = number of non-informative paralogous hit, PLOT = possible loci on the tip of the query genome contigs.
