# Supplementary material for: Impact of wet-lab protocols on quality of whole-genome short-read sequences from foodborne microbial pathogens
Source: Front Microbiol. 2023 Nov 29;14:1253362. doi: 10.3389/fmicb.2023.1253362 (PMC10716212; doi:10.3389/fmicb.2023.1253362)
Supplement: Supplementary Data Sheet 1 — Supplementary figures 1-4. [file Data_Sheet_1.zip › 1253362_Malorny_Data_Sheet_1.DOCX]

Supplementary Figures


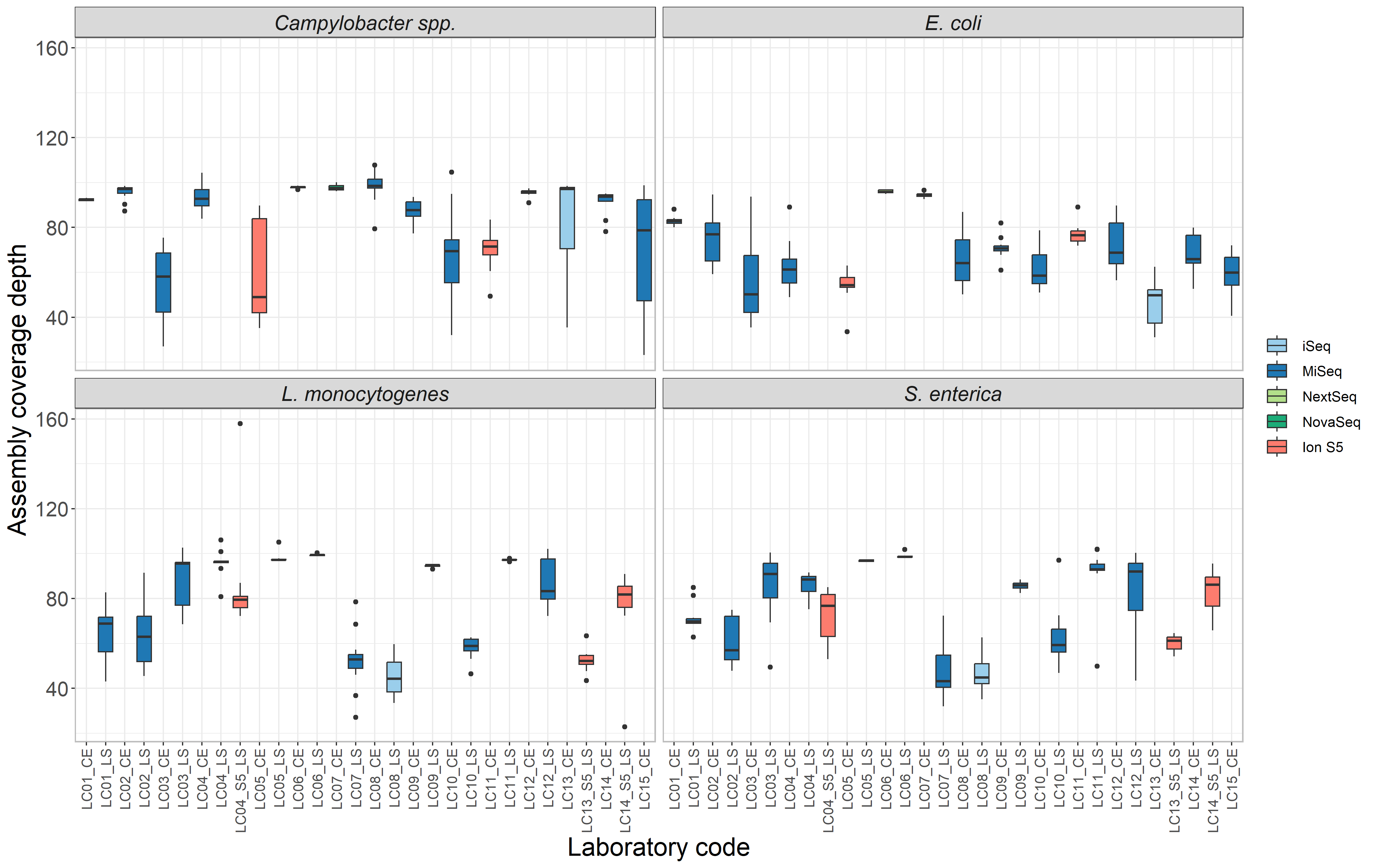
**Supplementary Figure 1.** Assembly coverage depth of all isolates per participant.

**
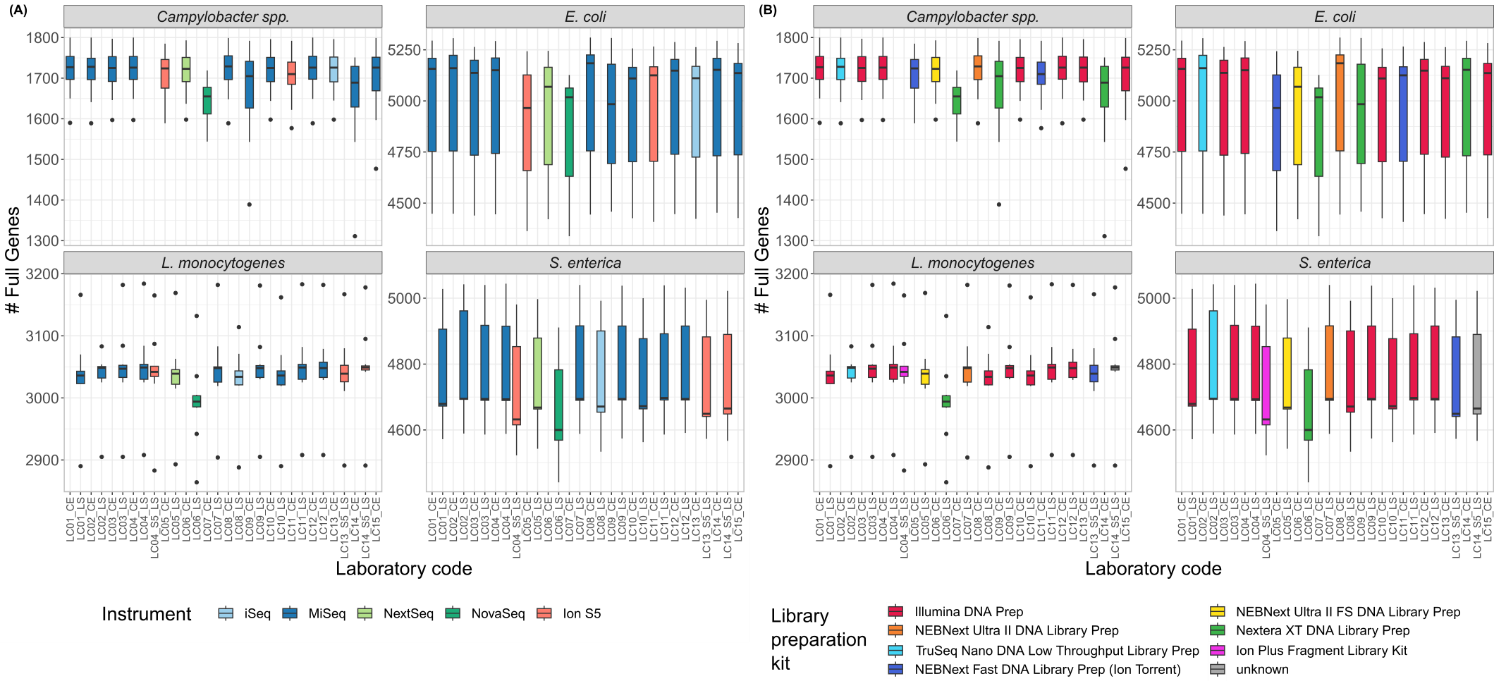
**

**Supplementary Figure 2.** Number of full genes in the assembly. Coloured per A) sequencing platform or B) applied library preparation kit.


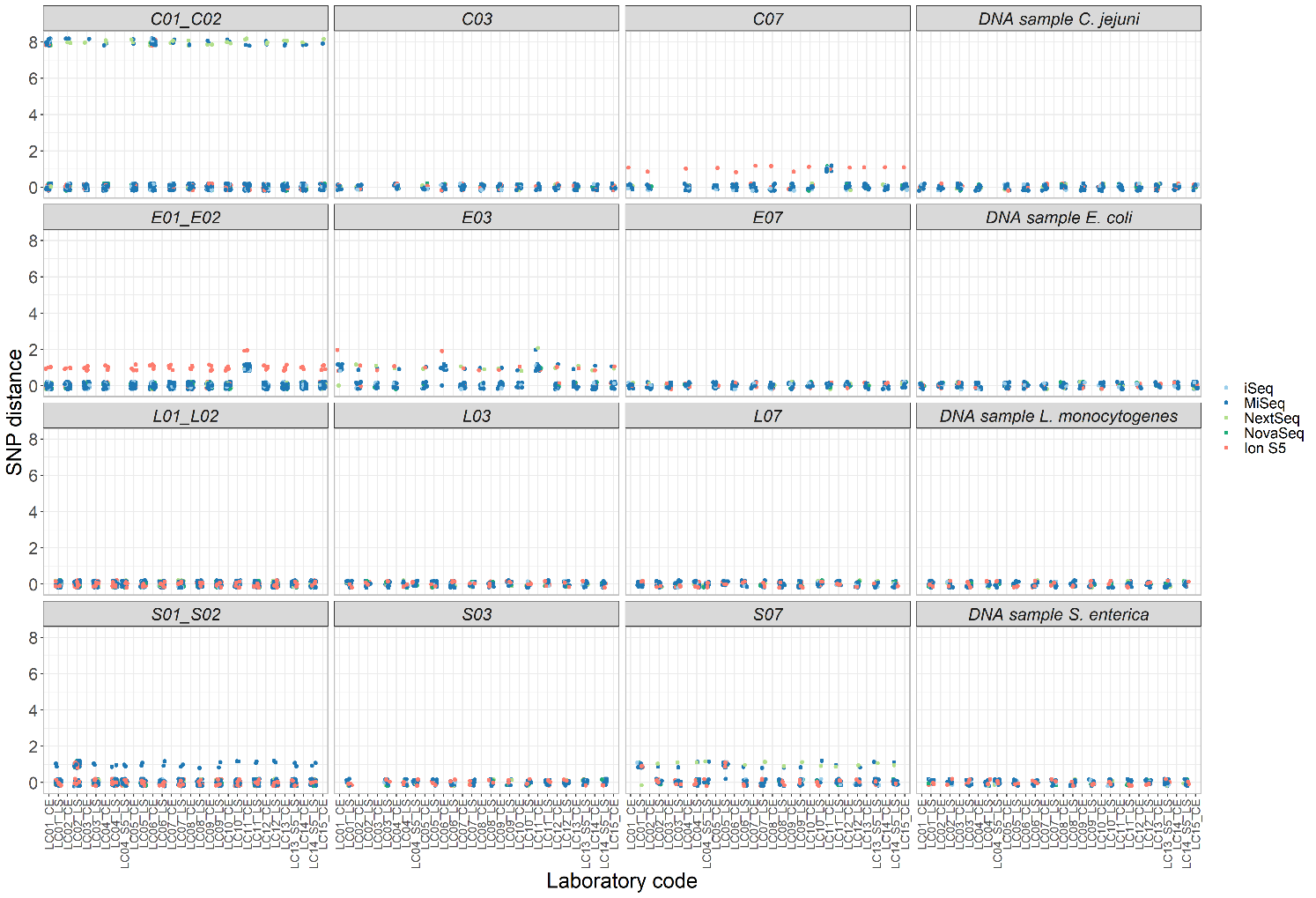


**Supplementary Figure 3.** Scatterplot of the pairwise SNP distances based on the participants data. Selected ring trial isolates were analyzed in individual SNP analyses with exception of the duplicated isolate (X01_X02) that were analyzed together.


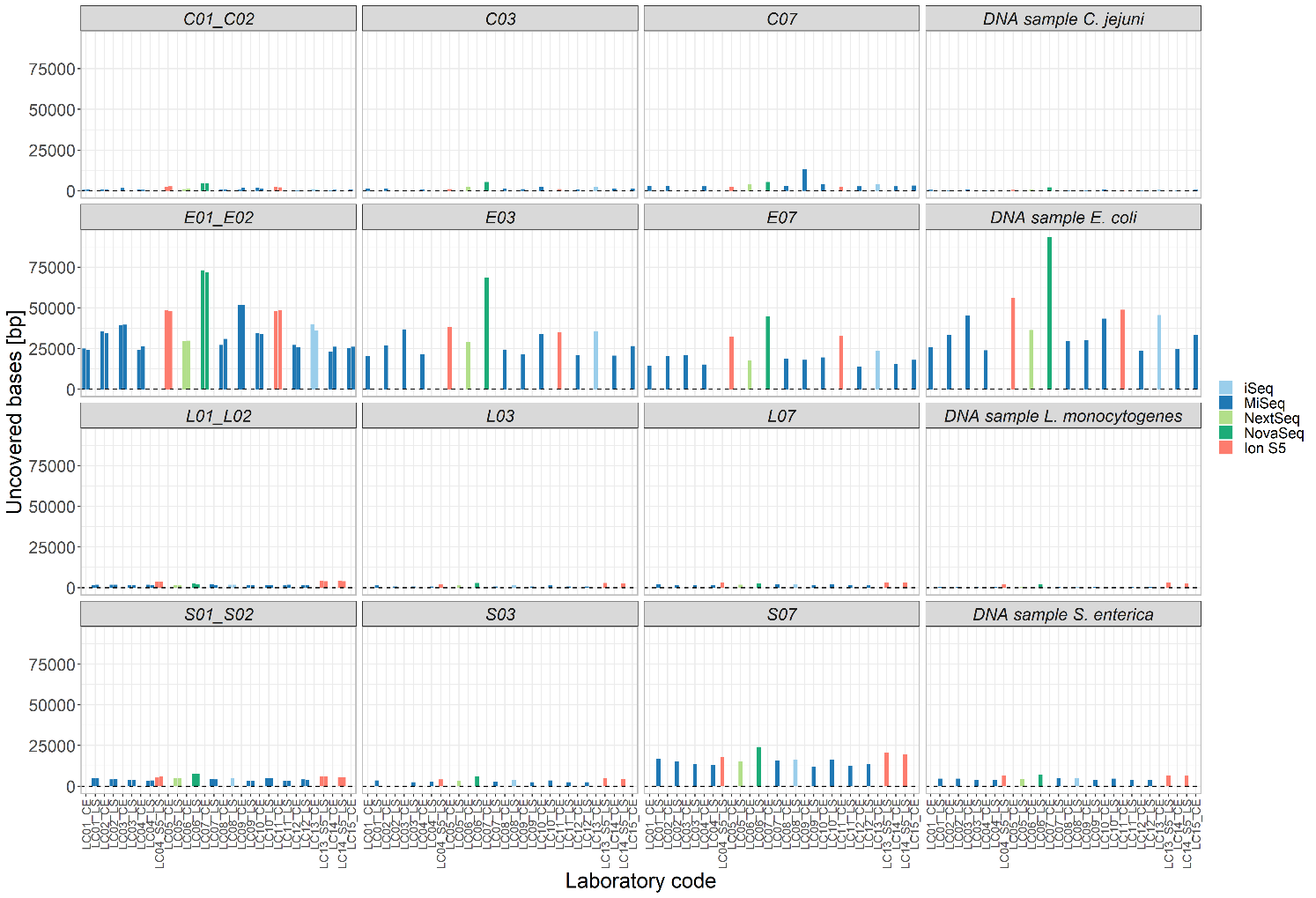


**Supplementary Figure 4.** Uncovered bases in the reference for SNP analysis per participant and analysis. Selected ring trial isolates were analyzed in individual SNP analyses with exception of the duplicated isolate (X01_X02) that were analyzed together.
